# Update on prevalence of *Babesia canis* and *Rickettsia* spp. in adult and juvenile *Dermacentor reticulatus* ticks in the area of Poland (2016–2018)

**DOI:** 10.1038/s41598-022-09419-y

**Published:** 2022-04-06

**Authors:** Dorota Dwużnik-Szarek, Ewa Julia Mierzejewska, Dorota Kiewra, Aleksandra Czułowska, Anna Robak, Anna Bajer

**Affiliations:** 1 Department of Eco-Epidemiology of Parasitic Diseases, Institute of Developmental Biology and Biomedical Sciences, Faculty of Biology, University of Warsaw, Miecznikowa 1, 02-096 Warsaw, Poland; 2 Department of Microbial Ecology and Acaroentomology, Faculty of Biological Sciences, University of Wrocław, Przybyszewskiego 63, 51-148 Wrocław, Poland

**Keywords:** Environmental sciences, Risk factors

## Abstract

Ornate dog tick, *Dermacentor reticulatus* is an important vector of *Babesia canis,* and *Rickettsia* spp. and other pathogens of veterinary and public health interest. The current study is the first to investigate the long-term changes in prevalence of these pathogens in expanding tick populations in Central Europe. Molecular techniques (PCR, sequencing) were applied for the detection of pathogen DNA in adult (n = 2497) and juvenile ticks (1096 larvae and 410 nymphs). DNA of *Rickettsia* spp. was identified in 35% of adults and 12.6% of juvenile ticks. DNA of *B. canis* was detected in 3% of adult ticks and only in ticks from the Eastern region (regional prevalence 6%). As previously, no *B. canis*-positive ticks were found in Western Poland, including ticks from Wrocław area (n = 298). DNA of *B. canis* was identified in 0.33% of juvenile ticks (in 3 pools of larvae and 2 nymphs) from the Eastern region. In the current study we confirmed high occurrence of *R. raoultii* in adults ticks from all four zones and relatively high prevalence of *B. canis* in the Eastern population of *D. reticulatus*, corresponding well with high incidence of canine babesiosis in this area of Poland. Finally, we confirmed *R. raoultii* and *B. canis* infection in all life stages of *D. reticulatus* ticks.

## Introduction

Vector-borne diseases constitute a serious problem of medical and veterinary importance^1,2^. Among canine tick-borne diseases, canine babesiosis, caused by apicomplexan parasite *Babesia canis* is of the greatest significance in central and eastern Europe^3^. Ornate dog tick, *Dermacentor reticulatus* is a main if not the only vector of *B. canis*^4^.

Recent study on the distribution of canine babesiosis in Poland in 2018 and occurrence of adult ticks in endemic areas and areas historically free of this tick species (2016–2018) revealed great discrepancy both in distribution of babesiosis cases and tick abundance, with ‘hot spot’ in Central and Eastern Poland^5^. The present study aimed to investigate the prevalence of *B. canis* in questing adult ticks collected from these regions and to find the link with distribution of babesiosis in Poland. Furthermore, *D. reticulatus* ticks are example of fast-spreading tick species and these dynamic changes in geographical range may be accompanied by changes in prevalence of pathogens in ticks. As we have previously determined prevalence of *B. canis* and *Rickettsia* spp. in ticks collected in years 2012–2014 from different regions of Poland^6^, the present study provides the update on prevalence of these pathogens in previously examined regions/sites and let us compare the long-term changes in infection frequency.

It is worth to underline, that in the previous studies *B. canis* was not detected in adult questing ticks from Western Poland, neither in juvenile ticks collected from rodents^6–8^ despite sporadic occurrence of canine babesiosis in this region^5^. In the current study we attempted to collect and examine a significant number of questing *D. reticulatus* ticks from Western Poland, including ticks collected in Wrocław area, in location with confirmed babesiosis cases in dogs.

Juvenile *D. reticulatus* ticks are heavily understudied in comparison to adult ticks, due to their hidden nidiculous style of life^1^. One of the aims of our current study was to examine a significant number of larvae and nymphs collected from rodents in the Eastern region of *D. reticulatus* occurrence for the presence of *B. canis* and *Rickettsia* spp. DNA and to compare prevalence of these pathogens between adult and juvenile ticks from the same sites.

Transovarial transmission of *B. canis* in *D. reticulatus* is believed to constitute the main route of transmission in vector population enabling persistence of parasite in certain tick populations^9–11^. Despite this belief, only few studies have confirmed the occurrence of *B. canis* in larvae and nymphs of ornate dog tick^12^. We have confirmed transovarial transmission of *B. canis* from infected females collected from dogs to eggs and larvae hatched in laboratory condition^11^. In present study we searched for *Babesia* spp. in juvenile ticks obtained from rodents, mainly voles. Detection of *B. canis* DNA in larvae and nymphs feeding on rodents (main reservoir of *B. microti*) would prove the efficiency of vertical transmission of *B. canis*. We hypothesize that an effective transovarial and transstadial transmission of *B. canis* in ticks are the main cause for its persistence in *D. reticulatus* population in *B. canis* hyperendemic areas in Central and Eastern Poland^5,13^.

*Dermacentor reticulatus* ticks are also important vectors of bacteria form the genus *Rickettsia*^14,15^. *Rickettsia* spp. transmitted by *D. reticulatus* are etiological agents of TIBOLA/DEBONEL (Tick-Borne Lymphadenopathy/Dermacentor-Borne-Necrosis-Erythema-Lymphadenopathy) group of diseases^16^. The first case of TIBOLA/DEBONEL in Poland was recorded in in 2011^17^. In our previous study (2012–2014), similar to *B. canis*, prevalence of *Rickettsia* spp. in *D. reticulatus* markedly differed in different regions of Poland^6^. Interestingly, the pattern was reverted, with much higher prevalence of *Rickettsia raoultii* in ticks collected from the Western endemic zone of *D. reticulatus* occurrence in comparison to Central and Eastern Poland^6^. The present study enabled the comparison of *Rickettsia* prevalence and distribution between two study periods and between adult and juvenile ticks collected from the Eastern endemic areas.

To summarize, the aims of the present study were: (1) to determine and compare the prevalence of *B. canis* and *Rickettsia* spp. in questing adult *D. reticulatus* ticks collected from different regions of Poland; (2) to determine the long-term trends in prevalence between different ticks populations; (3) to determine and compare prevalence of infection in juvenile *D. reticulatus* ticks in comparison to adult ticks from the same areas.

## Materials and methods

### Adult ticks

In the main study, adult questing ticks were collected in three-year period, 2016–2018, including 864 ticks from 2016, 877 from 2017 and 756 from 2018.

**Figure 1:**
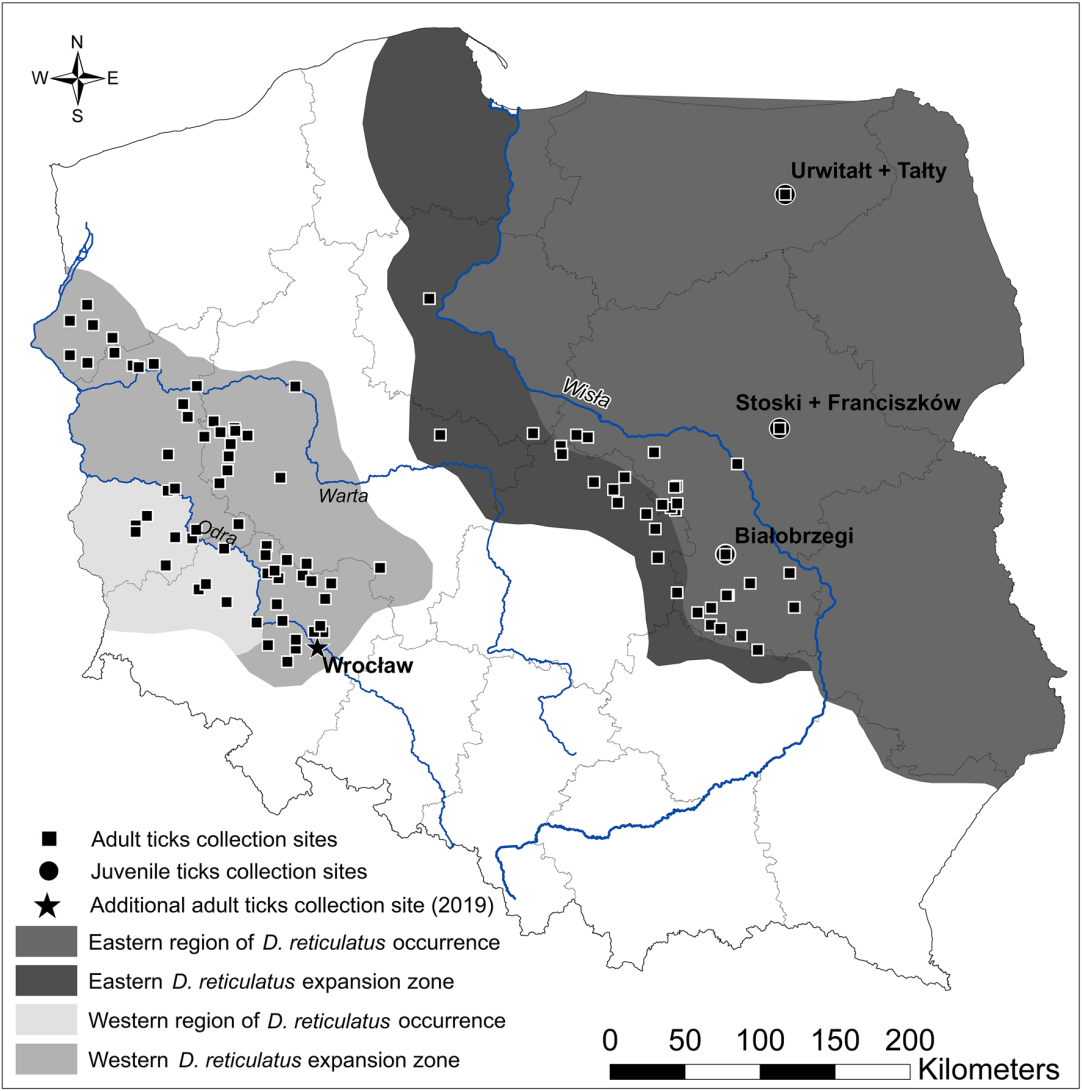
Map of endemic areas and expansion zones for adult questing *Dermacentor reticulatus* ticks (Dwużnik-Szarek et al. 2021) and sites of the collection of juvenile ticks. The map was designed using ArcGIS (ESRI) version 10.8.1 software (institutional licence purchased by the University of Warsaw, Warsaw, Poland). Briefly, each georeferenced location of tick collection (listed in Dwużnik-Szarek et al.^5^) was projected as a point type .shp layer and then used as the raw data for spatial analyses. A radius buffer was calculated for each point, allowing to interpolate a range of occurrence of the tick. The base layer consisted of contour map of Poland: country borders and largest administrative units (voivodeships).

In the main study, 2497 adult ticks (1446 females and 1051 males) were examined for pathogen occurrence: 1264 ticks from the Eastern region of *D. reticulatus* occurrence (1142 from the eastern endemic area and 122 from the eastern expansion zone) and 1233 ticks from the Western region of *D. reticulatus* occurrence (635 from the western endemic area and 598 from the western expansion zone) (zones marked in Fig. 1, following Dwużnik-Szarek et al.^5^ ).

All procedures of adult *D. reticulatus* collection were described in details in Dwużnik-Szarek et al.^5^.

Additionally, 298 questing adult *D. reticulatus* (152 females, 146 males) collected in Wrocław area in 2019, located within the city where cases of canine babesiosis were recorded (Kiewra, unpublished), were also examined.

### Larvae and nymphs collected from rodents

Altogether, 1096 larvae in 150 pools and 410 nymphs processed individually, were examined for *Rickettsia* spp. and *B. canis*. Description of collection of juvenile feeding ticks is provided in Dwużnik-Szarek et al.^8^ Briefly, rodents were trapped in habitats preferable for *D. reticulatus* (meadows, fallow lands and wetlands, etc.) in four sites in the Eastern region of *D. reticulatus* occurrence, endemic for *D. reticulatus*^5,8^ (Fig. 1): three sites in Mazovia voivodeship: Stoski + Franciszków (analyzed together as they are situated in close proximity of a few kilometers and represent similar open submerged habitat) and Białobrzegi and one site in Warmia-Mazuria voivodeship- Urwitałt (coordinates in Dwużnik-Szarek et al.^5^). Additionally, data on *Babesia* and *Rickettsia* occurrence in juvenile *D. reticulatus* collected from Białobrzegi and Urwitałt + Tałty (these sites were also analyzed together, for their close proximity and habitat similarity [mixed forest])^12^ was included in statistical analyses of prevalence.

### DNA isolation

Genomic DNA was extracted from ticks (larvae, nymphs, adults) using the Genomic Mini AX Tissue Spin kit (A&A Biotechnology, Gdańsk, Poland) in accordance with the manufacturer's protocol.

Adult *D. reticulatus* collected from the environment were processed individually. SPEX SamplePrep Freezer/Mill 6875D (Laboratory Equipment for Sample Preparation & Handling, Rickmansworth, Great Britain) was used to prepare the tick tissue homogenate for the subsequent extraction. This equipment enables the complete homogenization of 24 samples in the temperature of liquid nitrogen (− 195.8 °C) within 2 min. During one cycle, tick was placed in cryogenic vial and frozen in liquid nitrogen. Then it was pulverized into a homogeneous mixture with a magnetically driven impactor moved at great speed from one end to the other end of the vial. After the cycle was completed and the samples were removed from the grinder, the content of the vials was rinsed and transferred into 1.5 ml Eppendorf tubes with LSU buffer (Genomic Mini AX Tissue Spin kit, A&A Biotechnology, Gdańsk, Poland).

Juvenile ticks were drained of ethanol and then homogenized with a Tissue Grinder Mixy Professional homogenizer (Nippon Genetics Europe, Düren, Germany). From each rodent a maximum of 50 larvae (5 pools, up to 10 larvae in one pool) and 5 nymphs (processed individually) were tested.

### Pathogen detection

For detection of *Rickettsia* spp., primers Cs409 (5′-CCTATGGCTATTATGCTTGC-3′) and Rp1258 (5′-ATTGCAAAAAGTACAGTGAACA-3′) were used for amplification of a 750 bp fragment of the citrate synthase (*gltA*) gene^18^ as follows: initial denaturation in 95 °C for 5 min, 40 cycles of denaturation at a temperature of 95 °C for 30 s, 45 s of primer annealing in 59 °C, and elongation in 65 °C for 1 min^19^.

For molecular screening of *Babesia* spp., primers CRYPTO R (5'-GCTTGATCCTTCTGCAGGTTCACCTAC-3') and CRYPTO F (5'-AACCTGGTTGATCCTGCCAGT-3') were used to amplify ~ 1200 bp fragment of 18S rDNA in the first step of nested-PCR reaction. In second reaction primers BabGR2 (5'-CCAAAGACTTTGATTTCTCTC-3') and BabGF2 (5'-GYYTTGTAATTGGAATGATGG-3') amplified ~ 550 bp fragment of 18S rDNA^20^. Reaction conditions were as described in Tołkacz et al.^21^. For species-specific detection of *B. canis*, primers BcCOX1nR (5'-GGCCCTGTTCGGTATTGCAT-3') and BcCOX1nF (5'-CCATTTTGTTCTTTCAATTGGTGC-3') were used to amplify ~ 328 bp fragment of mitochondrial cytochrome c oxidase subunit 1 (*cox*1) gene^22^*.* Reaction conditions were as follows: 94 °C for 5 min, followed by 40 cycles at 94 °C for 20 s, 59 °C for 30 s, 72 °C for 45 s and final elongation at 72 °C for 7 minutes^22^. DNA of *B. canis* was used as positive control, negative controls were performed with 2 μl of sterile water in the absence of template DNA. PCR products were visualized on 1.5% agarose gel stained with Midori Green Stain (Nippon Genetics Europe, Düren, Germany).

Selected PCR products were sequenced by a private company (Genomed, Warsaw, Poland). Sequence alignments were carried out using BLAST-NCBI. Molecular phylogenetic analyses were performed in Molecular Evolutionary Genetics Analysis (MEGA) X open access software (https://www.megasoftware.net/) using Maximum Likelihood method of tree-construction. The evolutionary model was chosen with accordance to the data (following implemented model test in MEGA X) and bootstrapped over 1000 randomly generated sample trees.

### Statistical analysis

Minimum Infection Rate (MIR) was calculated for pools of larvae; if a sample was positive it was assumed that only one tick specimen in the pool was infected^19^. Analyses regarding larvae were conducted on MIR.

For the analysis of prevalence (% PCR-positive ticks), we applied maximum likelihood techniques based on log linear analysis of contingency tables in the IBM SPSS Statistics: PS IMAGO PRO Academic v.7 (institutional licence purchased by the University of Warsaw, Warsaw, Poland)^5,8^.

For adult ticks factors such a tick sex (two levels: male, female), region of *D. reticulatus* occurrence (two levels: the Western region, the Eastern region,), zones (four levels: western endemic zone, western expansion zone, eastern endemic zone, western expansion zone), season (two levels: spring, autumn), year (three levels: 2016, 2017, 2018) were used in models with the presence or absence of pathogen DNA (*B. canis*, *Rickettsia* spp.) considered as a binary factor (0, 1). In case of juvenile *D. reticulatus* ticks, sites (three levels: Białobrzegi, Franciszków + Stoski, Urwitałt + Tałty) and tick stage (larva or nymph) were used in models with the presence or absence of pathogen DNA (*B. canis*, *Rickettsia* spp.) considered as a binary factor (0, 1).

For each level of analysis in turn, beginning with the most complex model, involving all possible main effects and interactions, those combinations that did not contribute significantly to explaining variation in the data were eliminated in a stepwise fashion beginning with the highest level interaction (backward selection procedure). A minimum sufficient model was then obtained, for which the likelihood ratio of χ^2^ was not significant, indicating that the model was sufficient in explaining the data^5,8^. Values of P < 0.05 were considered as significant.

### Ethics approval

All of the procedures (trapping and handling of free-living rodents) were conducted with the approval of the First Warsaw Local Ethics Committee for Animal Experimentation in Poland (ethical license number: 706/2015), according to the principles governing experimental conditions and care of laboratory animals required by the European Union and the Polish Law on Animal Protection. Following collection of ticks, animals were released at the point of capture.

## Results

### Adult questing *Dermacentor reticulatus*

In total, 36.1% of examined ticks were infected with at least one pathogen. In the Western region of *D. reticulatus* occurrence (western endemic area + western expansion zone) 33.5% of ticks were infected, in the Eastern region (eastern endemic area + eastern expansion zone) 38.7% were infected (pathogen presence/absence × region of *D. reticulatus* occurrence: χ^2^_1_ = 7.30, P = 0.007).

*Babesia canis* was detected in adult ticks with total prevalence of 3.0% (74/2497) and as we suspected, DNA of *B. canis* was detected only in ticks from the Eastern region of *D. reticulatus* occurrence (*Babesia* presence/absence × region of *D. reticulatus* occurrence: χ^2^_1_ = 105.81, P < 0.0001), with regional prevalence of 5.9% (Table 1). No *B. canis*-positive ticks were found among 298 ticks collected from Wrocław area.

**Table 1:** Prevalence of *B. canis* and *R. raoultii* in *D. reticulatus* ticks in four zones.

Region (no. of ticks)	*Babesia canis*	95%Cl	*Rickettsia raoultii*	95%Cl	Zones	*Babesia canis*	95%Cl	*Rickettsia raoultii*	95%Cl
Eastern (1264)	5.9% (74/1264)	4.7–7.3	35.9% (454/1246)	33.3–38.6	Eastern endemic zone (1142)	6.1% (70/1142)	4.9–7.6	35.7% (408/1142)	33.0–38.5
					Eastern expansion zone (122)	3.3% (4/122)	1.1–7.6	37.7% (46/122)	29.5–46.5
Western (1233)	0	nc	33.5% (413/1233)	30.9–36.2	Western endemic zone (635)	0	nc	30.7% (195/635)	27.2–34.4
					Western expansion zone (598)	0	nc	36.5% (218/598)	32.7–40.4

Nc not calculated.

Slight difference in prevalence of *B. canis* was also observed between two eastern zones with about 6% of infected ticks in the eastern endemic area in comparison to about 3% in eastern expansion zone (not significant; NS) (Table 1).

Additionally, higher percentage of *B. canis*-positive ticks was noted in spring than in autumn (6.8% [95 Cl%: 5.1–8.8] vs 4.7% [95 Cl%: 3.2–6.7], respectively) (*Babesia* presence/absence × season: χ^2^_1_ = 23.60, P < 0.001). The highest prevalence of *B. canis* was noted in 2018 (8.5% [5.9–11.9%]), followed by 2017 (5.6% [3.9–7.9%]) and 2016 (4.0% [2.4–6.2%]) (*Babesia* presence/absence × year of tick collection: χ^2^_2_ = 6.99, P = 0.03). Tick sex had no effect on prevalence of *B. canis* (NS).

DNA of *Rickettsia* spp. was detected in 34.7% of total ticks. Prevalence of *Rickettsia* spp. was similar in the Eastern and Western region of *D. reticulatus* occurrence (Table 1, NS). There were some minor differences in percentage of PCR-positive ticks between four zones. Higher prevalence of *Rickettsia* was detected in ticks collected in the both expansion zones in comparison to the endemic regions (Table 1, NS). Season of tick collection and tick sex had no effect on prevalence of *Rickettsia* (NS). Interestingly, we detected significant differences in prevalence of this pathogen between years of tick collection. The highest prevalence, 39.4% [36.1–42.6%] was noticed in 2016 followed by 35.8% [32.7–39.0%] in 2017 and 28.2% [25.1–31.5%] in 2018 (*Rickettsia* presence/absence × year: χ^2^_2_ = 23.26, P < 0.001).

Co-infections of *Babesia* and *Rickettsia* could be recorded only in ticks collected in the Eastern region of the *D. reticulatus* occurrence, and only 3.2% (40/1264) of ticks from this region carried two pathogens (co-infection × region of *D. reticulatus* occurrence: χ^2^_1_ = 59.15, P < 0.0001).

### Juvenile *Dermacentor reticulatus*

Total prevalence of pathogens in juvenile *D. reticulatus*, including MIR, was 12.6% [11.1–14.3%]. Among instars, 8.7% [7.1–10.3%] of larvae and 23.4% [19.5–27.6%] of nymphs were positive for at least one pathogen (pathogen presence/absence × tick stage: χ^2^_1_ = 53.04 P < 0.001). Total prevalence by site (L + N) was the highest in Urwitałt + Tałty, Masuria—19.2% [15.6–23.3%], followed by Stoski + Franciszków in Mazovia—16.2% [10.4–23.7%], with the lowest value in Białobrzegi (Mazovia)—9.6% [7.8–11.5%] (pathogen presence/absence × site: χ^2^_2_ = 24.38, P < 0.001).

### *Rickettsia* spp.

12.6% [11.0–14.3%] of examined juvenile *D. reticulatus* ticks were *Rickettsia*-positive. Prevalence of *Rickettsia* was more than twice higher in nymphs compared to larvae (22.9% [19.1–27.2%] vs 8.7% [MIR] [7.1–10.4%], respectively) (*Rickettsia* present/absence × tick stage: χ^2^_1_ = 50.1, P < 0.001).

Among sites, the highest prevalence of this pathogen was detected in juvenile ticks in Urwitałt + Tałty, 19.0% [15.4–23.0%], followed by Stoski + Franciszków, 16.2% [10.4–23.2%] and Białobrzegi, 9.5% [7.8–11.4%] (*Rickettsia* presence/absence × site: χ^2^_2_ = 24.03, P < 0.001).

### *Babesia canis*

DNA of *B. canis* was identified in 0.33% [0.13–0.73%] of instars, with 0.27% [0.08–0.73%] in larvae (3 positive pools collected from *Microtus oeconomus*, *Myodes glareolus* and *Apodemus agrarius* in Białobrzegi) and in 0.49% [0.10–0.53%] of nymphs (one positive nymph collected from *M. oeconomus* in Białobrzegi, one nymph removed from *M. glareolus*, Urwitałt) (NS). DNA of *B. canis* was detected in juvenile ticks from Białobrzegi and Urwitałt + Tałty but not in much lower number of instars originated from Stoski and Franciszków (NS) (Table 2).

**Table 2:** Comparison of *B. canis* prevalence between juvenile and questing adult ticks *D. reticulatus* in endemic sites.

Site	Larvae	95% Cl	Nymphs	95% Cl	Adults	95% Cl
Białobrzegi	0.35% (3/859)	0.1–0.9	0.81% (1/124)	0.09–3.7	1.85% (1/54)	0.2–8.3
Stoski*	0% (0/52)	0	0% (0/65)	0	3.28% (8/244)	1.6–6.1
Urwitałt**	0% (0/185)	0	0.45% (1/221)	0.05–2.1	5.56% (8/144)	2.7–10.2
Total	0.27% (3/1096)	0.08–0.7	0.49% (2/410)	0.10–0.53	3.85% (17/442)	2.3–5.6

*Stoski + Franciszków together for juvenile tick stages.

**Urwitałt + Tałty together for juvenile tick stages.

### Comparison of *B. canis* prevalence between juvenile ticks and questing adult ticks collected from endemic areas

442 adult ticks from three endemic sites, Urwitałt, Białobrzegi and Stoski were examined for *B. canis* presence. DNA of *B. canis* was detected in all tick stages (in larvae, nymph and adult ticks) in Białobrzegi site (Table 2). Although there were some differences in prevalence between sites, they were insignificant (NS; Table 2).

### Molecular identification of pathogen species/genotypes

#### *Rickettsia* spp.

**Figure 2:**
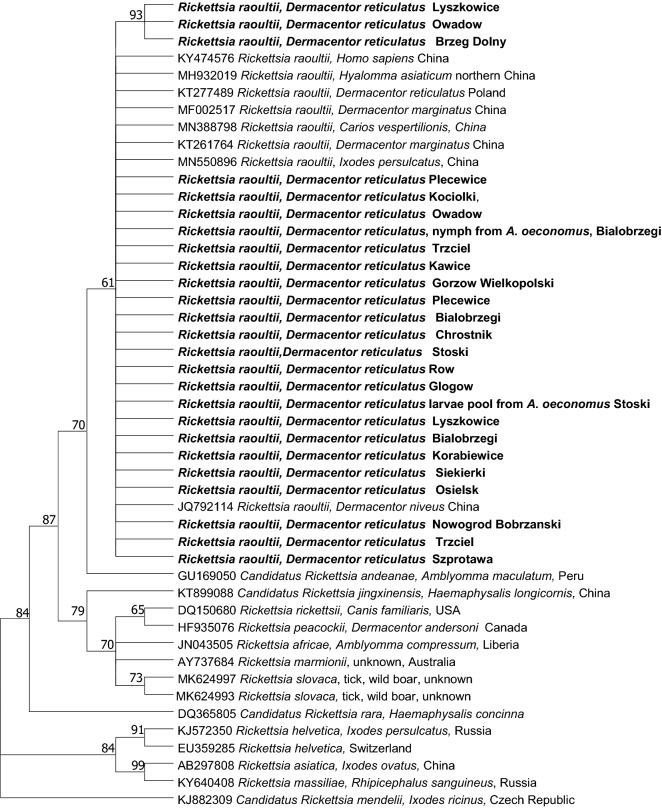
Molecular phylogenetic analysis of a 750 bp fragment of the citrate synthase (*gltA*) gene of *Rickettsia raoultii*. The evolutionary history was inferred by using the Maximum Likelihood method and Hasegawa-Kishino-Yano model. The tree with the highest log likelihood (− 1924.77) is shown. The percentage of trees in which the associated taxa clustered together is shown next to the branches. Initial tree(s) for the heuristic search were obtained automatically by applying Neighbor-Join and BioNJ algorithms to a matrix of pairwise distances estimated using the Maximum Composite Likelihood (MCL) approach, and then selecting the topology with superior log likelihood value. A discrete Gamma distribution was used to model evolutionary rate differences among sites (5 categories (+G, parameter = 0.3308)). This analysis involved 58 nucleotide sequences. There were a total of 703 positions in the final dataset.

42 of 867 *Rickettsia*-positive PCR products were sequenced. Among these, 38 were obtained from adult questing ticks (14 originated from eastern endemic area, four from eastern expansion zone, nine from western endemic area and 11 from western expansion zone). Four sequences were derived from juvenile *D. reticulatus* ticks: three sequences were obtained from larvae (Stoski) and one from a nymph (Białobrzegi). All obtained sequences displayed the highest identity (99.83–100%) to *R. raoultii* (GenBank accession numbers: MN388798 and MN550896). The phylogenetic tree, incorporating 25 sequences obtained in this study and 22 reference sequences from GenBank, is presented in Fig. 2. The tree topology showed that sequences obtained from examined ticks clustered on the one separate branch, as expected from BLAST analysis, constituting the *R. raoultii* clade.

#### *Babesia* spp.

**Figure 3:**
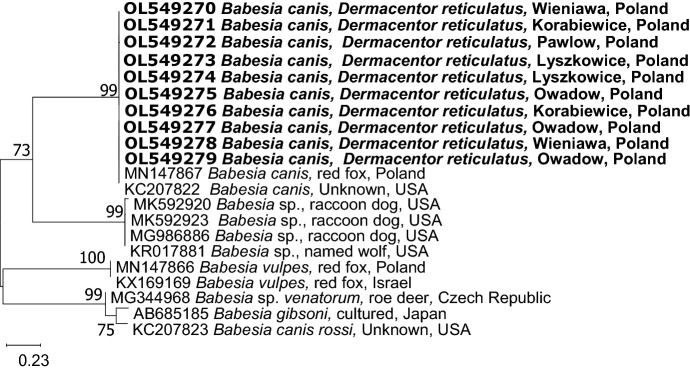
Molecular phylogenetic analysis of *cox1* of *Babesia* spp. (328 bp). The evolutionary history was inferred by using the Maximum Likelihood method and Hasegawa-Kishino-Yano model. The tree with the highest log likelihood (− 1413.72) is shown. The percentage of trees in which the associated taxa clustered together is shown next to the branches. Initial tree(s) for the heuristic search were obtained automatically by applying Neighbor-Join and BioNJ algorithms to a matrix of pairwise distances estimated using the Maximum Composite Likelihood (MCL) approach, and then selecting the topology with superior log likelihood value. The tree is drawn to scale, with branch lengths measured in the number of substitutions per site. This analysis involved 21 nucleotide sequences. There were a total of 236 positions in the final dataset.

Among 74 PCR products from adult *D. reticulatus* ticks, 31 *Babesia*-positive samples were sequenced. Sequences representing *cox*1 gene (n = 26) showed high similarity (in range 99.7–100%) to the sequence of *B. canis* derived from red fox from Poland (MN147867) and *B. canis* derived from a dog, USA (KC207822). Three sequences were derived from ticks collected in the eastern expansion zone and 22 from the eastern endemic region of *D. reticulatus* occurrence, Masovian voivodeship and one from Urwitałt, Warmia-Mazuria voivodeship. A representative tree for *cox*1 sequences (ten sequences derived from this study and 11 reference sequences from GenBank), obtained using the Maximum Likelihood method and Hasegawa-Kishino-Yano model is presented in Fig. 3. Our sequences (GenBank accession numbers OL549270- OL549279) clustered on one separate branch with the other two *B. canis cox*1 gene sequences deposited in GenBank.

Five PCR products of *Babesia* detected in adult ticks, representing 550 bp-fragment of 18S rDNA, were sequenced successfully. Representative sequence was deposited in GenBank under accession number MZ363934 and showed 99.8% identity (488/489) to *B. canis* derived from red fox (Poland), domestic dog (China) and *D. reticulatus* tick (Kazakhstan) (GenBank accession numbers MN134074, MK571831 and MK070118, respectively).

Additionally, two of three PCR products of *Babesia-*positive larvae (*cox*1 fragment) were sequenced successfully. Both samples were essentially identical (identity above 99%) to the sequence of *B. canis,* MN147867, from red fox, Poland.

## Discussion

Our current study allowed to monitor long-term dynamic of prevalence of two main pathogens vectored by *D. reticulatus* in endemic areas and zones of expansion in Central Europe. The main finding is stable great difference in prevalence of *B. canis* between the Western and Eastern populations of *D. reticulatus*. Higher prevalence of *B. canis* was also recorded in both endemic areas in comparison to both expansion zones. Additionally, there was the association between the occurrence of *B. canis* in adult questing ticks and juvenile stages in the eastern endemic sites of *D. reticulatus* presence. Interestingly, marked changes were observed in *R. raoultii* prevalence in ticks from different regions.

In the current study we found *B. canis*-positive ticks again only in the Eastern region of *D. reticulatus* occurrence with relatively high (about 6%) prevalence in the eastern endemic zone and half lower (3.3%) prevalence in the eastern expansion zone. The highest prevalence was noted in 2018 (8.5%). Similar pattern was found in our previous study^6^, with 8% prevalence in the Masovian endemic zone and almost 5% prevalence in the eastern expansion zone, west of the Vistula River. This finding supports successful maintenance/circulation of *B. canis* in the Eastern *D. reticulatus* population. Stable high prevalence of *B. canis* observed in central and eastern Poland (including capitol city Warsaw) over the period of six years is also reflected in high number of canine babesiosis cases (n = 1532) and incidence of babesiosis (53/1000 dogs), noted in that region in 2018, and accompanied by relatively high fatality rate of 2.5%^5^. Furthermore, in some localities in this region, incidence reached up to 250 cases/1000 dogs in 2018, thus confirming maintenance of hyperendemic region for canine babesiosis^5,13,23^. It is worth to underline, that in the current study the highest prevalence of *B. canis* was observed in Urwitałt, in Warmia-Mazuria voivodeship, one of the oldest area known as endemic for *D. reticulatus*^5,24,25^*. Babesia canis* was also identified in numerous recent studies on adult *D. reticulatus* collected in Eastern and NE Poland with prevalence in range 0.6–7.3%^26–29^.

In agreement with our previous study, DNA of *B. canis* wasn’t detected in ticks collected from Western Poland, neither in current nor in other studies^6,7^. We haven’t found *B. canis* DNA in ticks collected from Wrocław area, despite occurrence of canine babesiosis in this city^5^. However, the annual number and incidence of canine babesiosis cases in several veterinary clinics from the area of Western Poland were extremely low in 2018- a total 19 cases/year and 0.4/1000 dogs, respectively, in comparison with central and eastern Poland and suggest very low local prevalence in ticks (< 0.1%) or very focal occurrence of infected *D. reticulatus* ticks. Focal occurrence of *B. canis*-infected ticks was reported earlier in Switzerland^30^ and the UK^31^.

Recently, high percentage of *B. canis*- positive *Ixodes ricinus* ticks was detected near Poznań^32^. However, these results require more investigations, as this high prevalence is not reflected in the number/incidence of babesiosis cases in this locality^5^ and is not confirmed by other studies on *I. ricinus* ticks^4^.

*Rickettsia raoultii* was the most common pathogen in both adult and juvenile ticks. Bacteria from this genus are known as intracellular endosymbionts of various invertebrates, including ticks^33^. Prevalence of *R. raoultii* in adult ticks in the current study was higher in comparison to data from Austria (14.9%)^34^, Romania (18%)^35^, Slovakia (22.3–27%)^36^ and Ukraine (28%)^37^. Prevalence was lower than prevalence of *Rickettsia* observed four years earlier, during our previous study (44.1%)^6^ and in other studies in Poland (in range 41–44%)^26,38^. In our previous study significant differences in prevalence of *Rickettsia* between western and eastern populations were observed^6^. Prevalence of *Rickettsia* was by 10% higher in the Western population of *D. reticulatus* than in the Eastern one. In the present study prevalence was similar across four zones and two regions. However, in previous study (2012–2014) great majority of ticks (n = 1993) originated from the Eastern region of *D. reticulatus* occurrence and only 592 from the Western one, but in the current study (2016–2018) the number of examined ticks from both regions was similar (1264 vs 1233). Interestingly, although DNA of *R. raoultii* was identified in larvae and nymphs of *D. reticulatus*, the pattern was typical for tick-borne pathogens acquired externally (by feeding) and transmitted transstadially, with growing prevalence from larvae, through nymphs to adults.

Despite high prevalence of these bacteria in *D. reticulatus* population, the impact of *R. raoultii* on tick fitness or feeding process has yet to be elucidated^39,40^. For humans many *Rickettsia* species, including *R. raoultii,* are considered as pathogenic^41^. Prevalence of rickettsioses determined by indirect immunofluorescent assay (IFA) in Poland in years 2006–2012 reached 2.7%^42^. In North-Eastern Poland, where both *I. ricinus* and *D. reticulatus* ticks are abundant, presence of anti-*Rickettsia* IgG antibodies was confirmed in 51% of foresters and 27% of farmers^43^. Although registration of rickettsioses cases is obligatory in Poland, less than half of detected cases are likely reported^42^.

In current study, we made the effort to sample both adult and juvenile ticks from the sites endemic for *D. reticulatus* and *B. canis*. *Babesia canis* was identified in juvenile ticks, three larval pools and two nymphs, from one of this endemic sites with prevalence below 1%, while prevalence in adult ticks ranged from 2 to 5.6%. In Białobrzegi and Stoski sites only recently classified as endemic^5^, prevalence was much lower than in old endemic sites. But in Białobrzegi, *B. canis* was detected in every tick stage. In agreement with our results, Dunaj et al.^28^ reported *B. canis* infection in nine nymphs of *D. reticulatus* from the eastern endemic region. Detection of DNA of *B. canis* in partially engorged small specimens might have been negatively affected by the presence of other babesiae (i.e. *B. microti*) or other parasites (i.e. *Hepatozoon*) in blood meal taken by instars^12^. Identification of *B. canis* in larvae and nymphs collected from rodents supports the predicted routes of circulation of this piroplasm in endemic *D. reticulatus* population through transovarial and transstadial transmission. The main animal reservoir of *B. canis* in Poland constitute domestic dogs, as these parasites were rarely recorded in free-living canids, red foxes and grey wolves^5,13,22,23,44–46^.

## Conclusions

In the present study we determined the prevalence of *B. canis* and *Rickettsia* spp. in questing adult *D. reticulatus* ticks collected from different regions in Poland. Furthermore, we confirmed high occurrence of *R. raoultii* in adult ticks from all four zones and relatively high prevalence of *B. canis* in the Eastern population of *D. reticulatus*, corresponding well with high incidence of canine babesiosis in this area of Poland. Interestingly, no *B. canis*-positve ticks were found again in Western Poland, including Wrocław area. Finally, we confirmed *R. raoultii* and *B. canis* infection in all life stages of *D. reticulatus* ticks.
